# Nicotinamide Mononucleotide (NMN) Improves the Senescence of Mouse Vascular Smooth Muscle Cells Induced by Ang II Through Activating p-AMPK/KLF4 Pathway

**DOI:** 10.3390/ph18040553

**Published:** 2025-04-09

**Authors:** Na Liang, Si Liu, Yan Wang, Linyao Ying, Keyi Zhang, Hao Li, Lin Xiao, Yuming Hu, Gang Luo

**Affiliations:** 1 Xiangya School of Public Health, Central South University, Changsha 410078, China; 236912047@csu.edu.cn (N.L.); 226911029@csu.edu.cn (S.L.); 226911039@csu.edu.cn (Y.W.); 226912059@csu.edu.cn (L.Y.); zhangky@csu.edu.cn (K.Z.); 236912060@csu.edu.cn (H.L.); xiaolinxl@csu.edu.cn (L.X.); 2 Hunan Provincial Center for Disease Control and Prevention, Changsha 410078, China; 3 Hunan Academy of Preventive Medicine, Changsha 410078, China

**Keywords:** nicotinamide mononucleotide, vascular smooth muscle cells, senescence, AMPK/KLF4/p16 pathway, Angiotensin II

## Abstract

**Background**: Vascular smooth muscle cells (VSMCs) senescence exacerbates vascular diseases like atherosclerosis and hypertension. Angiotensin II (Ang II) is a strong inducer of VSMCs senescence, causing vascular damage, though its exact mechanism is unclear. Nicotinamide mononucleotide (NMN), a NAD^+^ precursor, has gained attention for its anti-senescence potential, yet its role in inhibiting VSMCs senescence is not fully understood. **Methods**: This study assessed senescence markers, including β-galactosidase activity (SA-β-gal) and the senescence-associated secretory phenotype (SASP), in mouse VSMCs treated with Ang II alone or with NMN and relevant activators/inhibitors. **Results**: Compared to controls, SA-β-gal levels and SASP secretion significantly increased in Ang II-exposed cells. In contrast, NMN reduced the expression of both markers. NMN also reversed Ang II-induced VSMCs senescence by downregulating KLF4 and p16 through AMPK activation, which Ang II inhibited, while decreasing mRNA levels of key SASP components. The effects of the AMPK activator AICAR were similar to those of NMN, whereas the AMPK inhibitor Compound C negated NMN’s effects. **Conclusions**: In summary, NMN mitigates Ang II-induced mouse VSMCs senescence via the AMPK/KLF4/p16 pathway. This study underscores the anti-senescence effects of NMN on mouse VSMCs, supporting further exploration of its potential as a food supplement for preventing and treating vascular senescence.

## 1. Introduction

Senescence refers to the gradual decline in tissue and organ function with age, impairing the body’s stress resistance and leading to disease and death. Cardiovascular diseases (CVDs), cerebrovascular diseases, and cancer are the main age-related illnesses, with CVD being the top cause of death among the elderly worldwide [1]. Vascular senescence significantly contributes to age-related changes in various organs. As early as 1680, Thomas Sydenham highlighted its importance, stating, “you are as old as your arteries” [2]. The American Heart Association reports that vascular senescence is now a major risk factor for CVD [3].

Vascular smooth muscle cells (VSMCs) are essential for arterial flexibility and tension, significantly contributing to vascular remodeling and diseases like atherosclerosis. Premature senescence of VSMCs drives vascular senescence, as these cells become larger, flatter, and show increased β-galactosidase activity along with a secretory phenotype that releases proinflammatory factors [4]. This contributes to chronic inflammation, vascular dysfunction, and accelerating atherosclerosis, calcification, and remodeling [5]. Angiotensin II (Ang II), a key regulator of the renin–angiotensin system, induces hypertension and vascular inflammation, often used to model VSMCs senescence [6]. While targeting VSMCs senescence can slow vascular disease progression [7], current anti-senescence drugs like dasatinib and quercetin, which eliminate senescent cells via apoptosis, can cause side effects like autophagy and inflammation [8]. Safer, more effective therapies are urgently needed.

Nicotinamide adenine dinucleotide (NAD^+^) is essential for cellular energy metabolism and redox balance, impacting mitochondrial function, DNA repair, cell division, and protein signaling [9]. The theory that NAD^+^ depletion contributes to senescence suggests that declining NAD^+^ levels lead to senescence by causing mitochondrial dysfunction and DNA damage [10]. The disruption of NAD^+^ homeostasis is associated with CVD [11], and NAD^+^ metabolic disorders recognized as modifiable cardiovascular risk factors [9]. Additionally, AMP-activated protein kinase (AMPK) inhibits cardiomyocyte senescence [12], with the NAD^+^/AMPK pathway offering cardiac protection by regulating PGC-1 and inhibiting mTOR [13]. Therefore, the NAD^+^/AMPK pathway is critical in cardiac senescence.

AMPK is vital for maintaining cellular energy homeostasis [14]. When ATP levels drop, AMPK is activated and phosphorylated, facilitating energy recovery [15]. Metabolomic analysis comparing healthy individuals and patients with aortic aneurysm and dissection showed significant AMPK enrichment in the healthy group, while another study linked the AMPK pathway to vascular senescence in diabetes [16]. Additionally, the absence of AMPK accelerates senescence in mouse fibroblasts by increasing p16 expression [17]. However, its role in regulating VSMCs senescence remains uncertain.

Kruppel-like factor 4 (KLF4), a key transcription factor regulating autophagy, is crucial for VSMCs phenotypic transformation [18]. Studies have shown that KLF4 plays a significant role in senescence and cardiovascular disease, with its expression fluctuating in mouse vascular senescence models, indicating its involvement in VSMCs regulation [19]. KLF4 knockdown significantly reduces AMPK-induced VSMCs transformation, causing growth arrest and the downregulation of SMC markers, confirming KLF4 as an AMPK target. Given the connection between NAD^+^ and senescence [20], it is hypothesized that NAD^+^ regulates AMPK and KLF4 in VSMCs senescence, presenting a potential therapeutic target for vascular senescence.

Nicotinamide mononucleotide (NMN), a key NAD^+^ precursor in mammals [21], has been shown to boost blood NAD^+^ levels, reducing oxidative stress (OS) and inflammation associated with senescence [22]. Studies reveal NMN extends lifespan, delays senescence, and improves vascular function in prematurely senescent mice [23], while addressing mitochondrial dysfunction and OS in elderly mice, and reducing cellular senescence in mesenchymal stem cells [24]. However, its effect on VSMCs function through intracellular NAD^+^ elevation remains underexplored, with studies limited to organ-on-a-chip models based on human aortic smooth muscle cells [25]. Among anti-senescence agents, NMN stands out due to its ability to directly raise NAD^+^ and its safety profile. We hypothesize that NMN could regulate AMPK and KLF4 in VSMCs senescence by increasing NAD^+^ levels.

## 2. Results

### 2.1. NMN Improved the Degree of Ang II-Induced Mouse VSMCs Cell Senescence and Reduced the Secretion of Key Senescence-Associated Secretory Phenotype (SASP) Factors

**Figure 1:**
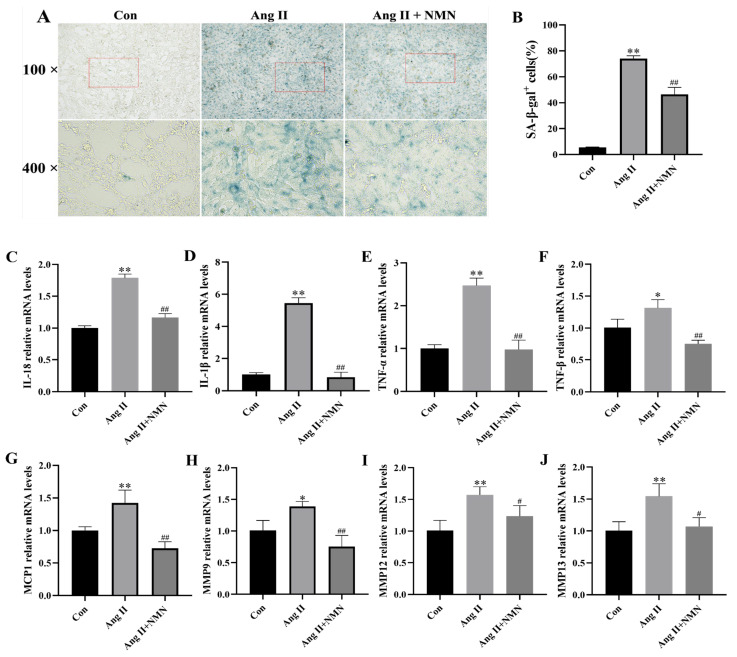
NMN improved the degree of Ang II-induced mouse VSMCs cell senescence and reduced the expression of key SASP factor transcription levels. (**A**,**B**) Representative images of SA-β-gal staining and quantitative analyses of SA-β-gal-positive cells (scale, 50 μm). (**C**–**J**) qRT-PCR analysis of IL-18, IL-1β, TNF-α, TNF-β, MCP-1, MMP9, MMP12, and MMP13 in mouse VSMCs. Data are expressed as the mean ± SD (n = 3, representing three independent experiments). * *p* < 0.05, ** *p* < 0.01 vs. the con group; ^#^ *p* < 0.05, ^##^ *p* < 0.01 vs. the Ang II group.

In order to assess the establishment of a senescence model of VSMCs stimulated by Ang II and the protective effect of NMN on VSMCs senescence, we compared SA-β-gal activity among the control group (con), Ang II group, and Ang II + NMN group. The secretion levels of SASP components in VSMCs were determined using qRT-PCR. The results from Figure 1A,B show that compared to the Con group, Ang II stimulation increased the number of SA-β-gal-positive cells in VSMCs, while NMN intervention significantly ameliorated the senescence status of VSMCs as evidenced by a decrease in the percentage of SA-β-gal-positive cells. Additionally, the qRT-PCR results (Figure 1) confirmed that following Ang II stimulation, mRNA levels for SASP components (Interleukin-18 (IL-18), Interleukin-1β (IL-1β), Tumor necrosis factor-α (TNF-α), Tumor necrosis factor-β (TNF-β), Monocyte chemotactic protein-1 (MCP-1), Matrix metalloproteinase-9 (MMP9), Matrix metalloproteinase-12 (MMP12), and Matrix metalloproteinase-13 (MMP13) were significantly higher than those in the Con group. However, NMN treatment led to the downregulation of these SASP components’ mRNA levels compared with those in the Ang II group.

### 2.2. NMN Regulates the AMPK/KLF4/p16 Pathway Associated with Senescence in Mouse VSMCs

**Figure 2:**
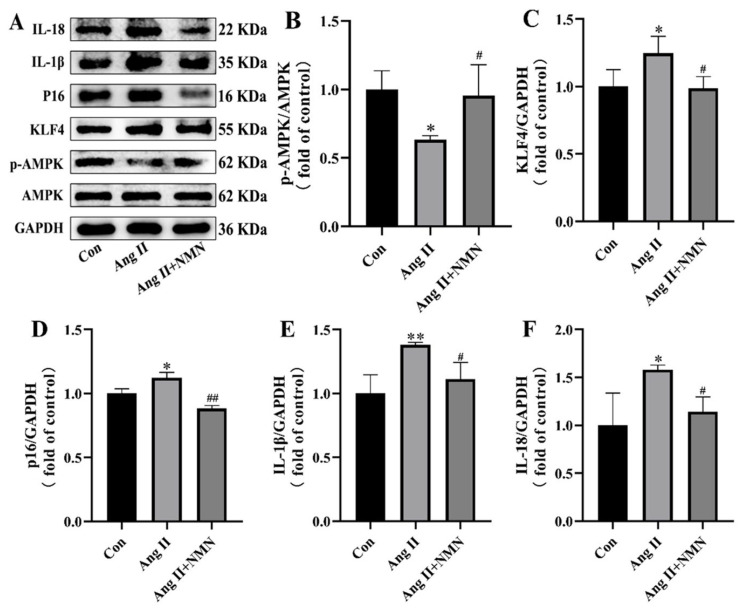
NMN regulates the AMPK/KLF4/p16 pathway associated with senescence in mouse VSMCs. (**A**) Representative Western blotting of AMPK, p-AMPK, KLF4, p16, IL-1β, IL-18, and GAPDH in mouse VSMCs. (**B**) p-AMPK/AMPK ratio. (**C**–**F**) Quantitative density analysis of KLF4, p16, IL-1β, and IL-18 normalized to GAPDH. Data are expressed as mean ± SD (n = 3, representing three independent experiments). * *p* < 0.05, ** *p* < 0.01 vs. the con group; ^#^ *p* < 0.05, ^##^ *p* < 0.01 vs. the Ang II group.

In order to determine whether Ang II and NMN affect mouse VSMCs cell senescence by regulating the AMPK/KLF4/p16 pathway, we detected AMPK phosphorylation levels and the expressions of KLF4, p16, IL-1β, and IL-18 by Western blot. As shown in Figure 2, AMPK phosphorylation was inhibited and the expressions of KLF4, p16, IL-1β, and IL-18 were increased in the Ang II group compared with the Con group. After the administration of NMN, AMPK phosphorylation was upregulated and the expression levels of KLF4, p16, IL-1β, and IL-18 were inhibited. These results suggest that NMN may inhibit the senescence of mouse VSMCs by regulating the activation of the AMPK/KLF4/p16 pathway.

### 2.3. AICAR Can Improve the Senescence of Mouse VSMCs Induced by Ang II and Reduce the Secretion of Key SASP Factors

**Figure 3:**
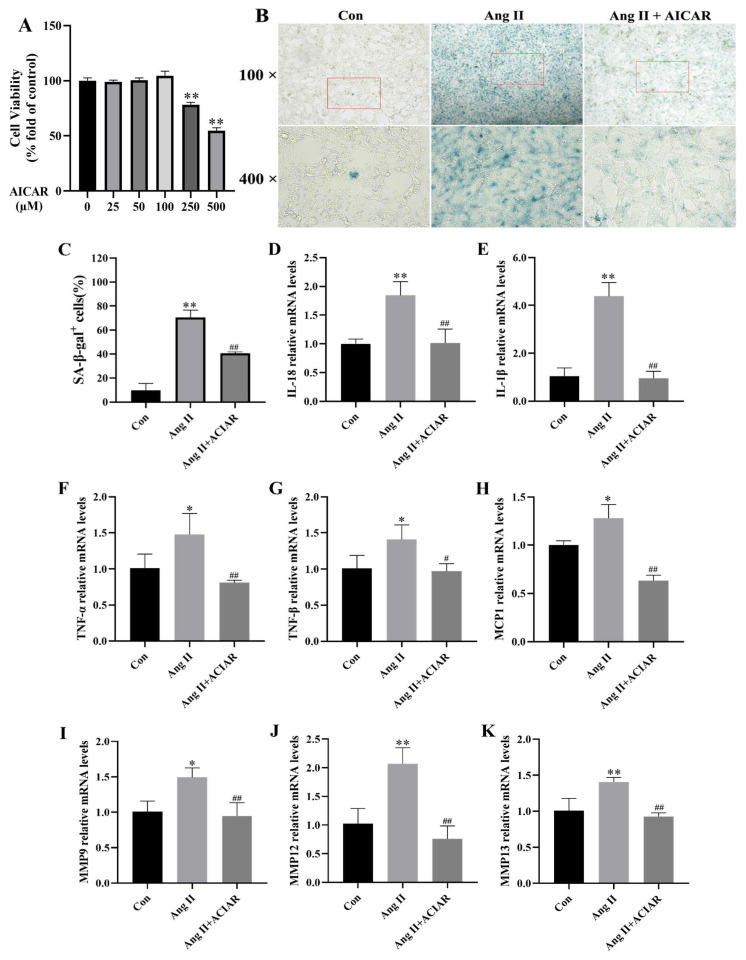
AICAR can improve the senescence of mouse VSMCs induced by Ang II and reduce the expression of key SASP factor transcription levels. (**A**) The effect of AICAR on the cell viability of mouse VSMCs. Data are expressed as mean ± SD (n = 3). (**B**,**C**) Representative images of SA-β-gal staining and quantitative analyses of SA-β-gal-positive cells (scale, 50 μm). (**D**–**K**) qRT-PCR analysis of IL-18, IL-1β, TNF-α, TNF-β, MCP-1, MMP9, MMP12, and MMP13 in mouse VSMCs. Data are expressed as the mean ± SD (n = 3, representing three independent experiments). * *p* < 0.05, ** *p* < 0.01 vs. the con group; ^#^ *p* < 0.05, ^##^ *p* < 0.01 vs. the Ang II group.

To further elucidate the crucial role of AMPK in Ang II-induced VSMCs senescence, we treated mouse VSMCs with Ang II alone or in combination with AICAR (a specific activator of AMPK). Initially, VSMCs were exposed to varying concentrations of AICAR for 24 h, and cell viability was assessed to determine the appropriate dose. Based on the CCK-8 results (Figure 3A), it was determined that AICAR at a concentration lower than 100 µM did not induce cytotoxicity in VSMCs; therefore, 100 µM AICAR was selected for subsequent experiments. The SA-β-gal staining results (Figure 3B,C) and qRT-PCR data (Figure 3D–K) demonstrated that treatment with 100 µM AICAR effectively mitigated Ang II-induced VSMCs senescence. The activation of AMPK reduced the proportion of SA-β-gal-positive cells and ameliorated aberrant mRNA levels of SASP components (IL-18, IL-1β, TNF-α, TNF-β, MCP-1, MMP9, MMP12, and MMP13) induced by Ang II.

### 2.4. The Activated AMPK/KLF4/p16 Pathway Is Involved in Ang II-Induced Mouse VSMCs Cell Senescence

**Figure 4:**
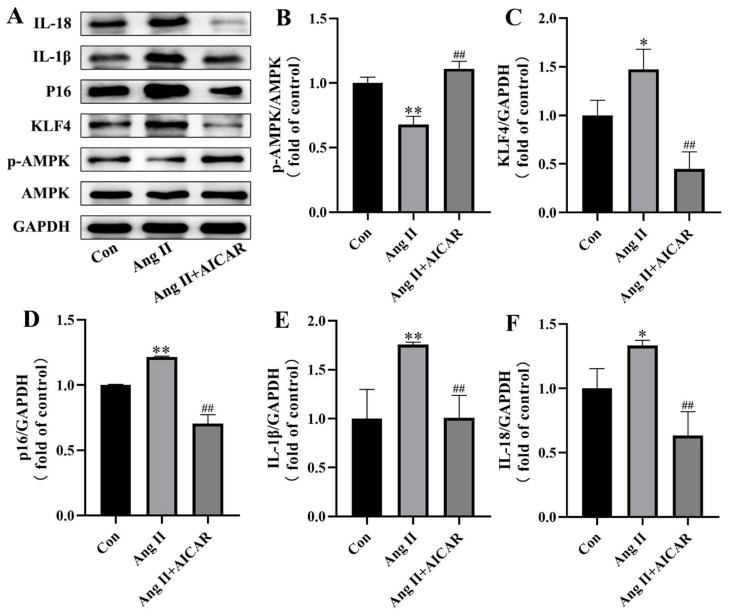
The activated AMPK/KLF4/p16 pathway is involved in Ang II-induced mouse VSMCs cell senescence. (**A**) Representative Western blotting of AMPK, p-AMPK, KLF4, p16, IL-1β, IL-18, and GAPDH in mouse VSMCs. (**B**) p-AMPK/AMPK ratio. (**C**–**F**) Quantitative density analysis of KLF4, p16, IL-1β, and IL-18 normalized to GAPDH. Data are expressed as mean ± SD (n = 3, representing three independent experiments). * *p* < 0.05, ** *p* < 0.01 vs. the con group; ^##^ *p* < 0.01 vs. the Ang II group.

To confirm the regulatory effect of Ang II on VSMCs cell senescence through the AMPK/KLF4/p16 pathway, we utilized a Western blot analysis to assess AMPK phosphorylation and the expression of KLF4, p16, IL-1β, and IL-18 in the con group, Ang II group, and Ang II + AICAR group. As depicted in Figure 4, Ang II treatment led to inhibited AMPK phosphorylation and increased expressions of KLF4, p16, IL-1β, and IL-18 compared with the con group. However, following treatment with an activator, not only was the inhibited AMPK phosphorylation level restored, but the expression of KLF4, p16, IL-1β, and IL-18 was also downregulated. These findings indicate that the AMPK/KLF4/p16 pathway plays a crucial role in Ang II-induced senescence of VSMCs and may represent a potential therapeutic target.

### 2.5. Compound C Could Mask the Effect of NMN on the Senescence of VSMCs Induced by Ang II

**Figure 5:**
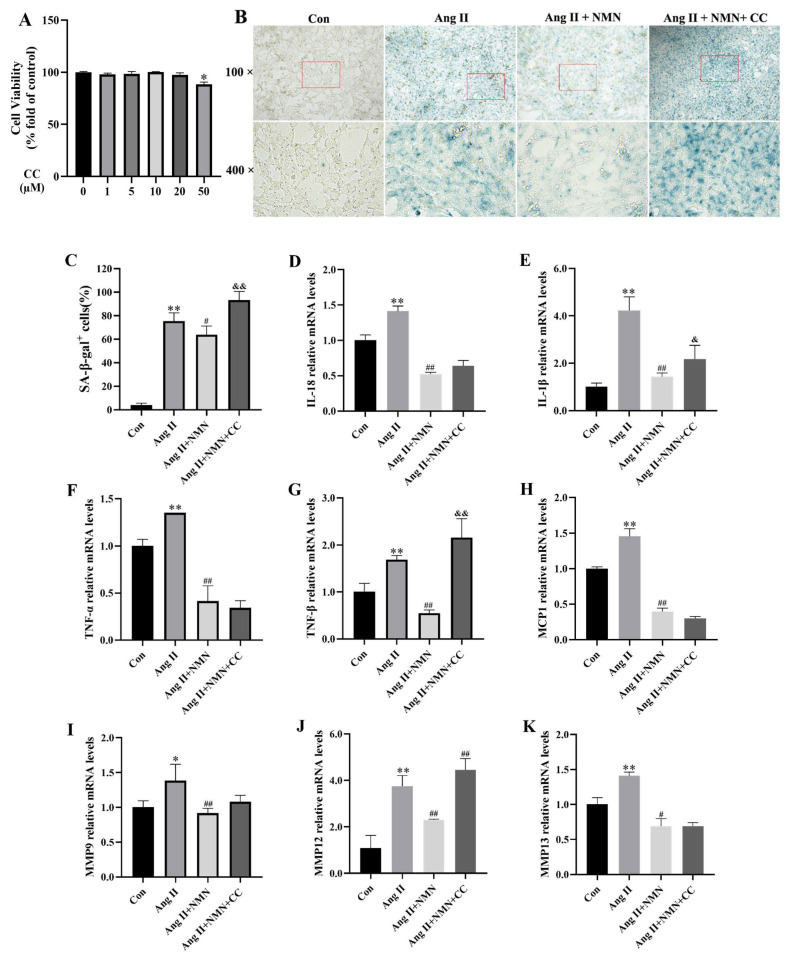
Compound C could mask the effect of NMN on the senescence of VSMCs induced by Ang II. (**A**) The effect of Compound C on the cell viability of mouse VSMCs. Data are expressed as mean ± SD (n = 3). (**B**,**C**) Representative images of SA-β-gal staining and quantitative analyses of SA-β-gal-positive cells (scale, 50 μm). (**D**–**K**) qRT-PCR analysis of IL-18, IL-1β, TNF-α, TNF-β, MCP-1, MMP9, MMP12, and MMP13 in mouse VSMCs. Data are expressed as the mean ± SD (n = 3, representing three independent experiments). * *p* < 0.05, ** *p* < 0.01 vs. the con group; ^#^ *p* < 0.05, ^##^ *p* < 0.01 vs. the Ang II group, ^&^ *p* < 0.05, ^&&^ *p* < 0.01 vs. the Ang II + NMN group.

In order to investigate whether AMPK is a crucial target of Ang II and NMN in the regulation of VSMCs cell senescence, we introduced a subgroup intervention with Ang II, NMN, and Compound C (an AMPK specific inhibitor) based on the previous experimental group. The viability of VSMCs treated with different concentrations of Compound C was assessed using the CCK-8 assay for 24 h. The results (Figure 5A) indicated that concentrations of Compound C below 20 µM did not induce cytotoxicity in VSMCs. Based on these findings and the CCK-8 results, a concentration of 10 µM Compound C was selected for subsequent experiments. SA-β-gal staining results (Figure 5B,C) demonstrated that after treatment with 10 µM Compound C, the proportion of SA-β-gal-positive cells in both the Ang II + NMN + Compound C group and Ang II group was similar, and both were higher than that in the Ang II + NMN group. The qRT-PCR analysis (Figure 5D–K) revealed that the mRNA levels of SASP components (IL-1β, TNF-β, and MMP12), which were initially restored by NMN, were subsequently further elevated upon AMPK inhibition, though they remained lower compared to the Ang II group. To conclude, 10 µM Compound C effectively suppressed AMPK phosphorylation in VSMCs and partially alleviated the impact of NMN on VSMCs cellular senescence.

### 2.6. Inhibition of AMPK/KLF4/p16 Pathway Increased the Senescence of Mouse VSMCs Induced by Ang II

**Figure 6:**
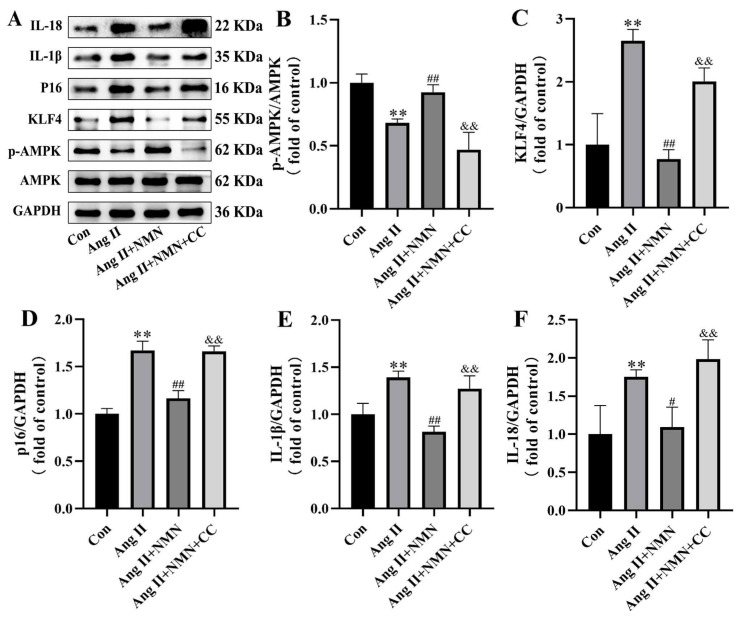
The AMPK inhibitor reversed the effects of NMN on Ang II-induced markers of senescence. (**A**) Representative Western blotting of AMPK, p-AMPK, KLF4, p16, IL-1β, IL-18, and GAPDH in mouse VSMCs. (**B**) p-AMPK/AMPK ratio. (**C**–**F**) Quantitative density analysis of KLF4, p16, IL-1β, and IL-18 normalized to GAPDH. Data are expressed as mean ± SD (n = 3, representing three independent experiments). ** *p* < 0.01 vs. the con group, ^#^ *p* < 0.05, ^##^ *p* < 0.01 vs. the Ang II group; ^&&^ *p* < 0.01 vs. the Ang II + NMN group.

We observed that NMN may impact the senescence process of VSMCs via the AMPK/KLF4/p16 pathway. In order to further validate the pivotal role of this pathway in the VSMCs cell senescence process, a Western blot analysis was employed to detect the con group, Ang II group, Ang II + NMN group, and Ang II + NMN + CC group levels of AMPK phosphorylation and expression levels of KLF4, p16, IL-1β, and IL-18. The results (Figure 6) showed that NMN could restore inhibited AMPK phosphorylation by Ang II and downregulate the expression of KLF4, p16, IL-1β, and IL-18. However, after Compound C intervention, the phosphorylation level of AMPK was almost completely suppressed, and the expression of KLF4, p16, IL-1β, and IL-18 was upregulated. These results suggest that NMN may regulate the senescence process of mouse VSMCs through AMPK/KLF4/p16.

## 3. Discussion

Besides age-related factors, vascular senescence involves various pathogenic mechanisms, among which the importance of VSMCs senescence has become increasingly prominent [26]. As a risk factor for various vascular diseases, Ang II has been shown to induce VSMCs senescence [27], but the specific mechanism is not fully understood. In this study, we investigated the senescence process of VSMCs induced by Ang II and the anti-senescence effect of NMN and its mechanism. We demonstrate for the first time that Ang II can trigger VSMCs senescence by inhibiting the AMPK/KLF4/p16 pathway. NMN supplementation reversed Ang II-induced VSMCs senescence by activating the AMPK/KLF4/p16 pathway.

The senescence of VSMCs plays a crucial role in the progression of AS and various CVDs, and intervening in the senescence process of VSMCs may be an important strategy for preventing and treating CVD [28,29]. Ang II regulates blood pressure and VSMCs proliferation [30], making it essential to understand its targets that trigger VSMCs senescence. Consistent with previous studies [27,31], there was a significant increase in the percentage of senescent SA-β-gal-positive cells in VSMCs after stimulation with Ang II as well as an increase in SASP factor levels. Our study observed the inhibition of AMPK phosphorylation during Ang II-induced VSMCs senescence, aligning with previous models of Ang II-induced hypertension, myocardial oxidative damage, aortic aneurysm, and dissection [16,32,33]. In addition, supplementation with AMPK activator AICAR was also effective in reversing Ang II-induced VSMCs senescence [16]. Previous studies have also demonstrated that the inhibition of AMPK’s involvement in Ang II-stimulated premature senescence of VSMCs [34] and deletion of AMPKα2 can upregulate p16 expression, collectively leading to cell senescence [17]. Hiroaki found that KLF4 is directly targeted by AMPK in VSMCs [19]; our study found that when Ang II inhibited AMPK, expression levels increased for KLF4/p16. A previous study suggested that the overexpression of KLF4 acts as a protective factor against Ang II-induced VSMCs senescence by enhancing autophagy; KLF4 expression increased short-term Ang II exposure (<4 weeks) and decreased after prolonged exposure (>4 weeks) in mouse blood vessels [27]. Another similar study found that short-term Ang II (<5 days) exposure significantly increased KLF5 expression in VSMCs, while chronic Ang II stimulation (>5 days) significantly reduced KLF5 expression levels [30]. In our study, which spanned 72 h, we did not observe a protective effect of KLF4, possibly due to the intervention’s duration. Given the multifaceted role that KLF4 may play at different times, caution should be exercised when interpreting its effects. Nonetheless, our results provide evidence of an upstream–downstream relationship between AMPK and KLF4 in VSMCs.

Senescent VSMCs secrete various SASP factors with pro-inflammatory properties, which contribute to reduced proliferation and migration, increased inflammation, phenotypic transformation, and calcification—processes that collectively accelerate cellular senescence [4]. Ang II induces OS in VSMCs, triggering an inflammatory response that produces signaling molecules such as TGF-β, MCP-1, and matrix metalloproteinases (MMPs) [35]. IL-1β and MCP-1 regulate VSMCs phenotypic transition and MMP production, with MMPs playing a key role in VSMCs growth and atherosclerosis. MMP-9 is essential for VSMCs migration, while both MMP-9 and MMP-12 cleave N-cadherin, influencing VSMCs proliferation and apoptosis [36]. Pro-inflammatory factors like IL-18 are elevated in senescent cells, driving inflammation and dysfunction. VSMCs senescence is critical in the pathophysiology of vascular diseases, and inhibiting it offers protective effects [8].

NMN is commonly found in everyday foods, and its positive impact on anti-senescence and the prevention of various age-related complications has been well-documented [37,38]. Studies have shown that supplementation with NMN can improve cerebral microvascular and neurovascular function, as well as reverse vascular dysfunction [39,40]. The regulation of NMN has a complex interaction with SASP [41]. However, there has been limited research on the effects of NMN on different types of vascular cells. In order to investigate its anti-senescence effects on VSMCs, a key component of blood vessels, we utilized NMN to intervene in VSMCs stimulated by Ang II. As anticipated, our findings showed that NMN supplementation effectively reversed Ang II-induced VSMCs senescence, significantly reducing both the percentage of SA-β-gal-positive senescent cells and the levels of SASP factors. These findings suggest that NMN may indeed represent a promising therapeutic agent for VSMCs senescence.

**Figure 7:**
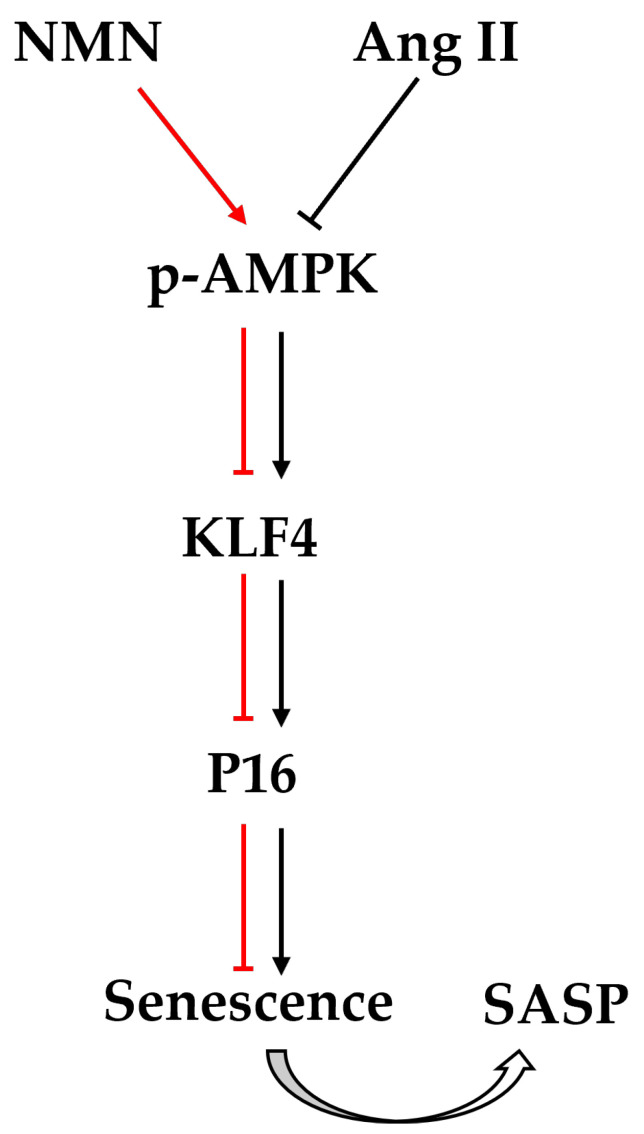
Mechanism of Ang II-induced senescence in mouse VSMCs Model. The red color represents the effect of NMN, while the black color indicates the effect of Ang II. Arrows signify activation, and horizontal lines represent inhibition.

To further investigate the anti-senescence mechanism of NMN in VSMCs, we conducted a study to examine the AMPK pathway protein. Our results demonstrated that NMN was able to restore the inhibited AMPK level caused by Ang II, downregulate the expression of KLF4/p16, and mitigate the senescence effect of Ang II on VSMCs. Additionally, supplementation with AMPK inhibitor Compound C was found to abolish the anti-senescence effect of NMN on VSMCs. Based on these findings, we propose that NMN exerts its anti-senescence role through the AMPK/KLF4/p16 signaling pathway (Figure 7). The physiological function of NMN in vivo primarily relies on adenylate transferase for its conversion into NAD^+^ [42]. The sirtuin enzyme family comprises seven NAD^+^-dependent protein deacetylases, which are well-known longevity factors [43]. NAD^+^ supplementation can directly restore AMPK activation or activate AMPK by maintaining SIRT1 deacetylation of serine/threonine kinase 11 [44,45]. It can also regulate AMPK phosphorylation by maintaining SIRT3 deacetylase activity [46]. Therefore, it is plausible that NMN may mediate the AMPK pathway to participate in VSMCs senescence through various signaling pathways involving NAD^+^.

In this study, we only investigated the effects of AMPK activators and inhibitors. Future studies should involve silencing and overexpressing various proteins in the AMPK/KLF4/p16 pathway to confirm their role in VSMCs senescence. Additionally, it should be noted that AICAR alone are not sufficient to completely reverse Ang II-induced VSMCs senescence. Similarly, the effect of compound C on NMN is also limited, suggesting that other potential mechanisms are involved and require further study.

## 4. Materials and Methods

### 4.1. Reagents and Antibodies

The following reagents were used: beta-Nicotinamide mononucleotide (purity: 99.08%, APExBIO, Boston, MA, USA), Ang II (purity: 99.98%, MCE, Monmouth Junction, NJ, USA), AICAR (APExBIO), and Compound C (APExBIO), SA-β-gal stain kit (Solarbio Technology, Beijing, China). Reagents for cell culture were purchased from Adamas (Titan Technology, Shanghai, China). All the other chemicals used were of reagent grade and purchased from commercial suppliers. The primary antibodies used for Western blotting were anti-AMPKa1/AMPKa2 (ABclonal Technology, Wuhan, China, A12718), anti-phospho-AMPKa1/AMPKa2-T183/T172 (ABclonal, AP1441), anti-KLF4 (Novus Biologicals, Littleton, CO, USA, NBP2-24749), anti-p16INK4a (Abcam, Cambridge, United Kingdom, ab211542), anti-IL-18 (Affinity Biosciences, Changzhou, China, DF6252), anti-IL-1β (Affinity, AF5103), and GAPDH (ABclonal, AC033). Horseradish peroxidase (HRP)-linked anti-rabbit IgG (Beyotime Biotechnology, Shanghai, China, A0208) and HRP-linked anti-mouse IgG (Beyotime, A0216) were also used.

### 4.2. Senescence-Galactosidase Staining (SA-β-Gal) Was Evaluated for Senescence

The cells were inoculated onto a six-well plate for culture and intervention. The SA-β-Gal kit was utilized to stain the cells as per the provided instructions. Following the observation of staining in both experimental and control cells, the percentage of SA-β-gal-positive areas within the visual field was determined with approximately equal numbers of cells. All the images were captured using a fluorescent cell imager (Thermo Fisher EVOSM7000, Waltham, MA, USA) and analyzed using Image-J 1.8.0. The light source was set to “Trans”, and the brightness mode was set to “Precision”.

### 4.3. Total RNA Isolation and Real-Time Quantitative Polymerase Chain Reaction (qRT-PCR)

According to the manufacturer’s protocol, total RNA extraction from cells was carried out using TransZol Up (Trans Gen Biotech, Beijing, China). Subsequently, cDNA synthesis was performed using 1 μg of total RNA and oligo-dT primers according to the manufacturer’s instructions and DNA contamination was eliminated (Trans Gen Biotech). The qRT-PCR reaction was conducted using Bright Cycle Universal SYBR Green qPCR Mix with UDG (ABclonal) kit on a Roche real-time fluorescence quantitative PCR system as per the manufacturer’s guidance, with GAPDH serving as the internal reference gene. Excel was used to summarize the data, and the 2^−∆∆CT^ method was employed to normalize the expression of the relevant genes. The primers were synthesized by Sangon Biotech (Shanghai, China), and their specific sequences are listed in Table 1.

### 4.4. Cell Culture and Treatment

The mouse aortic vascular muscle cell lines were purchased from American Type Culture Collection (CRL-2797, Manassas, VA, USA), and cultured in a 5% CO_2_ incubator at 37 °C using DMEM supplemented with 10% fetal bovine serum, 100 U/mL penicillin, and 100 µM streptococcus. To investigate the mechanism of Ang II-induced senescence in smooth muscle cells and the potential benefits of NMN, mouse VSMCs were divided into five groups: (1) Con group; (2) Ang II group: treated with 0.1 µM Ang II for 72 h; (3) Ang II + NMN group: treated with 0.1 µM Ang II for 48 h, followed by 0.1 µM Ang II + 0.5 mM NMN for 24 h; (4) Ang II + AICAR group: treated with 100 µM AICAR (AMPK activator) + 0.1 µM Ang II for 4 h, followed by 0.1 µM Ang II for 68 h; and (5) Ang II + NMN + Compound C group: treated with 0.1 µM Ang II for 48 h, followed by 0.1 µM Ang II + 0.5 mM NMN + 10 µM Compound C (AMPK inhibitor) for 24 h. Before each treatment, the medium of the same specification was replaced in all the groups, and the control group received an equal volume of medium as the intervention group for balancing. The dosages and durations of AICAR and Compound C were selected based on cell viability results and previous studies. These compounds were prepared as highly concentrated stock solutions, with only minimal working volumes (≤10 µL) added at a time, ensuring they remained non-cytotoxic to mouse VSMCs.

### 4.5. Cell Viability

After 24 h of treatment with different concentrations of AICAR (0, 25, 50, 100, 250, or 500 µM) and Compound C (0, 1, 5, 10, 20, or 50 µM), a Cell Counting Kit-8 (CCK-8, Eno Gene Biotech, Nanjing, China) was used to detect mouse VSMCs cell viability.

### 4.6. Western Blot Analysis

Cells were lysed by RIPA lysis buffer (Dingguo, Beijing, China) containing 1% phosphatase inhibitor (Beyotime) and 1% PMSF (Dingguo) to extract the total cell protein. The total proteins were quantified by a BCA (Beyotime) kit and diluted by 5×SDS-PAGE Loading buffer (Beyotime). The same amount of protein was separated using 10% SDS-PAGE and transferred to polyvinylidene fluoride (PVDF, Millipore Corp, Billerica, MA, USA) membrane by transmembrane solution. After sealing with skim milk, the primary antibody was incubated overnight (AMPK, 1:1000; p-AMPK, 1:1000; KLF4, 1:1000; p16, 1:1000; IL-1β, 1:1000; Il-18, 1:1000; and GAPDH, 1:30,000). The membranes were incubated with HRP-linked secondary antibody (1:8000) for one hour. The film was impregnated with ECL chemiluminescence reagent (Epizyme, Shanghai, China), and the bands were visualized in a Western Blotting image analyzer (Azure Biosystems, Dublin, CA, USA, Azure 600). Finally, Image-J was used to quantitatively analyze the protein bands.

### 4.7. Statistical Analysis

All experimental data were expressed as mean ± SD, and differences between groups were analyzed using ANOVA and LSD post hoc test in SPSS 26.0 software (IBM, Armonk, NY, USA). When *p* < 0.05, the results were considered statistically significant.

## 5. Conclusions

In summary, this study provides relevant evidence for Ang II-induced senescence of VSMCs and the anti-senescence effects and mechanisms of NMN. Ang II inhibited the AMPK/KLF4/p16 pathway and triggered VSMCs senescence. However, NMN reversed Ang II-induced senescence by restoring AMPK/KLF4/p16 activity. Therefore, by focusing on the anti-senescence properties of the AMPK/KLF4/p16 pathway in VSMCs, this study provides new insights into the protective ability of NMN against VSMCs senescence and offers a potential approach for further investigation in the therapeutic area dealing with new solutions in vascular protection.

## Figures and Tables

**Table 1:** Real-time quantitative PCR primer sequences.

Gene	Forward Primer 5′-3′	Reverse Primer 5′-3′
IL-18	ATAAATGACCAAGTTCTCTTCGTTGAC	CACAGCCAGTCCTCTTACTTCAC
IL-1β	TCGCAGCAGCACATCAACAAG	TCCACGGGAAAGACACAGGTAG
TNF-α	CACGCTCTTCTGTCTACTGAACTTC	CTTGGTGGTTTGTGAGTGTGAGG
TNF-β	TCTGGAGAGCAAGCACGGATC	ACCACCTGGGAGTAGACAAAGTAG
MCP-1	CACTCACCTGCTGCTACTCATTC	GCTTCTTTGGGACACCTGCTG
MMP9	AGTATCTGTATGGTCGTGGCTCTAAG	GGAGGTGCTGTCGGCTGTG
MMP12	GGCAACTGGACAACTCAACTCTG	CGCTTCATCCATCTTGACCTCTG
MMP13	CAGTTGACAGGCTCCGAGAAATG	CACATCAGGCACTCCACATCTTG
GAPDH	GCAAATTCAACGGCACAGTCAAG	TCGCTCCTGGAAGATGGTGATG

## Data Availability

The original contributions presented in this study are included in this article. Further inquiries can be directed to the corresponding authors.

## Abbreviations

The following abbreviations are used in this manuscript:

VSMCs	Vascular smooth muscle cells
NMN	Nicotinamide mononucleotide
Ang II	Angiotensin II
SASP	Senescence-associated secretory phenotype
SA-β-gal	Senescence-associated β-galactosidase
NAD^+^	Nicotinamide adenine dinucleotide
AMPK	AMP-activated protein kinase
KLF4	Kruppel-like factor 4
IL-18	Interleukin-18
IL-1β	Interleukin-1β
TNF-α	Tumor necrosis factor α
TNF-β	Tumor necrosis factor β
MCP-1	Monocyte chemotactic protein-1
MMP9	Matrix metalloproteinase-9
MMP12	Matrix metalloproteinase-12
MMP13	Matrix metalloproteinase-13
